# Prevention and Treatment of Esophageal Stenosis after Endoscopic Submucosal Dissection for Early Esophageal Cancer

**DOI:** 10.1155/2014/457101

**Published:** 2014-10-16

**Authors:** Jing Wen, Zhongsheng Lu, Qingsen Liu

**Affiliations:** ^1^Department of Gastroenterology and Hepatology, Chinese PLA General Hospital, No. 28 Fuxing Road, Haidian District, Beijing 100853, China; ^2^Department of Gastroenterology and Hepatology, Chinese PLA 261 Hospital, Shangzhuang Township, Haidian District, Beijing 100094, China

## Abstract

Endoscopic submucosal dissection (ESD) for the treatment of esophageal mucosal lesions is associated with a risk of esophageal stenosis, especially for near-circumferential or circumferential esophageal mucosal defects. Here, we review historic and modern studies on the prevention and treatment of esophageal stenosis after ESD. These methods include prevention via pharmacological treatment, endoscopic autologous cell transplantation, endoscopic esophageal dilatation, and stent placement. This short review will focus on direct prevention and treatment, which may help guide the way forward.

## 1. Introduction

Endoscopic submucosal dissection (ESD) of high-grade dysplasia and early esophageal cancer has gained acceptance in the last decade as an effective therapeutic option [1, 2]. However, the residual mucosal defect after the procedure may cause acute inflammation, deep ulcers, local submucosal fibrous connective tissue proliferation, collagen deposition, esophageal wall fibrosis, and even esophageal stricture formation [3]. The incidence of esophageal strictures after endoscopic resection for near-circumferential or circumferential esophageal large mucosal defects has been extremely high at 88–100% [4–9]. Dysphagia of varying degree is one of the most common symptoms of benign esophageal stenosis, whereas other clinical manifestations such as nausea, vomiting, weight loss, and even cachexia can also occur depending on the degree of stenosis. Patient quality of life is seriously affected due to these symptoms; thus, active prevention and treatment of esophageal stenosis are necessary.

Esophageal stenosis can be divided into simple or complex stenosis depending on the length, shape, and lumen diameter of the stenosis, and different types of esophageal stenosis respond differently to treatment. Simple stenosis refers to esophageal stenosis that is limited to a certain segment of the esophagus without obvious tortuosity of the esophageal lumen through which gastroscopy can still be performed [10, 11]. Complex stenosis refers to esophageal stenosis >2 cm with obvious tortuosity of the esophageal lumen or through which gastroscopy cannot be performed [10]. Most cicatricial stenoses caused by ESD are refractory esophagus stenosis [5] for which there is no efficient prevention or treatment, which presents a challenge. In this paper, we review studies on the prevention and treatment of esophageal stenosis that were published in the last decade to explore the research status and development direction of the prevention and treatment of esophageal stenosis after ESD.

## 2. Prevention of Esophageal Stenosis after ESD

### 2.1. Pharmacological Treatment

#### 2.1.1. Glucocorticoids

Glucocorticoids can inhibit inflammation and reduce the formation of fibrous connective tissue as a result of scar tissue softening [12, 13]. Local submucosal injection of glucocorticoids through endoscopy has been increasingly used in the treatment of refractory benign esophageal stenosis [14–16]. In a study of 41 patients, Hashimoto et al. [17] found a lower incidence of stenosis and a smaller number of patients needing balloon dilatation in the treatment group than in the control group, with no obvious complications. All 21 patients in the treatment group received an endoscopic shallow injection of triamcinolone at the base of the ulcer within 3 days after ESD at a total dosage of 2 mg/cm depending on the resection diameter. In the study by Hanaoka et al. [18], 30 patients were selected to receive an endoscopic injection of glucocorticoids immediately after ESD for early esophageal cancer to prevent esophageal stenosis, with a historical control of 29 patients who had previously undergone the esophageal ESD procedure. There were no significant differences in the parameters related to tumor size or range of lesion involvement between the two groups, but the incidence of stenosis and the frequency of balloon dilatation decreased in the glucocorticoid treatment group compared with that in the historical controls.

It was also widely reported that oral glucocorticoids could be used to prevent stenosis after ESD. In a study of 7 patients who underwent ESD for circumferential lesions, 4 patients in the glucocorticoids group began receiving oral prednisone on day 3 after ESD at dosages of 30 mg, 25 mg, 25 mg, 20 mg, 15 mg, 10 mg, and 5 mg for 7 days, with gradual reduction to withdrawal after 8 weeks. Endoscopic balloon dilatation (EBD) was performed when necessary if patient in the treatment group developed dysphagia. In contrast, patients in the control group underwent balloon dilatation twice a week for a total of 8 weeks from day 3 after ESD. The esophageal stenosis was ultimately dilated to 18 mm. Isomoto et al. [8] found that balloon dilatation was needed significantly less frequently in the treatment group than in the control group without any adverse effects. A consistent result was achieved by Yamaguchi et al. [9], in which the sample size was greatly increased to 41 patients. In a case report in the same year, Yamaguchi et al. [19] recorded that a patient who received preventive treatment with oral glucocorticoids after ESD for near-circumferential early esophageal cancer did not develop dysphagia or need balloon dilatation and that no adverse effect was observed. The difference in the route of administration of glucocorticoids to prevent esophageal stenosis was also reported. Sato et al. [20] reported that 23 patients who underwent complete circumferential ESD for superficial esophageal carcinoma were managed with EBD alone (*n* = 13) or with EBD and oral prednisolone (*n* = 10). Patients given steroids + EBD required fewer sessions and shorter management period than those in the EBD alone group. A total of 43 patients who underwent ESD for early esophageal cancer were randomized into 2 groups in the study of Mori et al. [21], and 23 patients underwent balloon dilatation combined with endoscopic injection of glucocorticoids and the other 20 patients underwent balloon dilatation combined with glucocorticoid gel. Although no significant difference in operation time was observed between the two groups, the frequency of balloon dilatation for dysphagia and the volume of bleeding during the operation were significantly different, which indicated that glucocorticoid gel was more effective and safer for preventing esophageal stenosis after ESD than endoscopic injection.

#### 2.1.2. Antineoplastic Drugs

Mitomycin C, an effective antineoplastic drug, can simultaneously inhibit fibroblast proliferation [22]. Mitomycin C has been widely used in the prevention and elimination of scars in fields including ophthalmology, plastic surgery, otolaryngology, urology, orthopedics, and upper gastrointestinal tract [22–24]. In a retrospective study of 5 patients who developed refractory esophageal stenosis after ESD and needed repeated balloon dilatation, Machida et al. [25] found no recurrence or drug adverse effect in the 4.8 months after the injection of mitomycin C at the site of dilatation subsequent to balloon dilatation.

5-Fluorouracil (5-FU) is a traditional antineoplastic drug that can inhibit cell proliferation by inhibiting DNA synthesis and adding RNA to interfere with protein synthesis. In recent years, 5-FU was reportedly used in the treatment of hypertrophic scars and cicatricial stenosis [26–28]. High-concentration 5-FU often leads to necrosis, whereas low-concentration 5-FU inhibits fibroblast proliferation, which reduces the formation of cicatricial tissues. Mizutani et al. [29] discovered a preventative effect of endoscopically injected 5-FU against esophageal stenosis after ESD in an animal model. 5-FU was also administered as collagen-coated liposomes that continued releasing 5-FU in the body of animals to maintain the drug concentration and reduce the injection frequency.

### 2.2. Endoscopic Cell Transplantation

Numerous studies have confirmed that autologous stromal cells promote the regeneration of organs and tissues, and this concept has already been applied to myocardial, vascular, skin, and nerve tissues. In a randomized controlled trial by Honda et al. [30], an animal model was established with 10 dogs. In the treatment group, 8 mL of cellular matrix suspension buffer was injected using endoscopy into the residual submucosa after esophageal ESD, which contained derived cellular matrix isolated from autologous adipose tissue. In contrast, the control group was injected with the same dosage of acellular matrix buffer. As a result, both the dysphagia scores and the degree of mucosal damage were lower in the treatment group, whereas the unit submucosal new microvascular number was higher in the treatment group than in the control group. Additionally, significant atrophy and fibrosis were observed in the esophageal muscularis propria in the control group compared with that in the treatment group. The trial's findings indicated that the injected autologous adipose matrix cells could inhibit contraction of the esophageal mucosa in the animal model of dogs, thus improving the clinical symptoms related to esophageal stenosis after ESD. In an animal model trial of Takagi et al. [31], transplantable oral mucosal epithelial cell sheets were fabricated from the patients' oral mucosa. After endoscopic mucosal resection (EMR) or ESD, the fabricated autologous cell sheets were endoscopically transplanted to the ulcer sites. However, the incidence of structure is not clearly described in the study. Kanai et al. [32] found that fabricated autologous skin epidermal cell sheets would be useful in preventing severe esophageal constriction after circumferential ESD. In that study, although all pigs in the control and transplanted groups showed severe esophageal constriction after 2 weeks, the weight gain and the mean degrees of constriction differed significantly. Early reepithelialization and mild fibrosis in the muscularis were observed in the transplanted group.

Later, an extraordinary article was published in* Gastroenterology* in 2012. Ohki et al. [33] collected samples of oral mucosal tissue from 9 patients with superficial esophageal squamous cell neoplasia, from which cells were isolated and cultured in vitro at an appropriate temperature to prepare epithelial sheets after 16 days. These epithelial sheets were transplanted through endoscopy to the surface of an ulcer after esophageal ESD, and endoscopic examination was conducted once a week until the epithelium was completely formed. The approximate time taken for the endoscopic epithelium reconstruction of the surface of ulcer was 3.5 weeks, and the procedure was successful in 8 patients without any incidence of dysphagia, stenosis, or other complications, and one patient with full circumferential ulceration underwent EBD 21 times. This promising finding undoubtedly broadened the way of thinking in the prevention of esophageal stenosis after ESD. Most recently, Hochberger et al. [34] reported that gastroesophageal mucosal transplantation for stricture prevention after widespread ESD for early cancers seemed feasible. In that study, after ESD for upper early esophageal cancer, the gastric antral mucosal specimen was cut into 3 pieces and attached to the mucosal defect by hemoclips and then fixed using an uncovered metal mesh stent that was removed on postprocedural day 20. Within 5 months after the procedure, the area of mucosal transplant had gradually grown nearly circumferential in the esophagus. However, the patient had a 1 cm, nonserious stricture formation but no other complaints.

## 3. Treatment of Esophageal Stenosis after ESD

### 3.1. Endoscopic Esophageal Dilatation

Endoscopic esophageal dilatation is an effective approach to treating benign esophageal stenosis [35]. Current endoscopic dilatation mainly includes bougienage and balloon dilatation. Bougienage can be divided into Maloney and Savary-Gilliard types depending on the bougie used. The bougie in Maloney bougienage is filled with mercury or tungsten, whereas that in Savary-Gilliard bougienage is made of polyvinyl compound and is guided using a guide wire. Balloon dilatation includes dilatation through X-ray fluoroscopy and dilatation through-the-scope (TTS). Among them, Savary-Gilliard bougienage and TTS balloon dilatation are the most common endoscopic dilatation approaches used for esophageal benign stenosis in clinical practice owing to their safety, convenience, and efficiency. In Savary-Gilliard bougienage, the guide wire is inserted into the stomach through the esophageal stenotic region from the gastroscopic biopsy channel and a bougie of appropriate diameter is then chosen depending on the degree of esophageal stenosis. Small to large bougies are selected and used to dilatate the stenosis step by step to an appropriate extent. As for TTS balloon dilatation, a balloon catheter is inserted through the stenotic region under endoscopy and then gas or liquid is injected into the balloon for distraction when the stenosis ring is positioned at the middle of the balloon. Dilatation was performed for 1–3 min depending on patient tolerance, and the balloon is deflated and withdrawn after completion of dilatation. In most studies, no significant difference in efficiency was found between the 2 dilatation approaches [36–38]. However, Savary-Gilliard bougies can be reused, whereas TTS balloons are used only once. Hence, Savary-Gilliard bougies are more economical.

Standard endoscopic dilatation with bougienage and balloon dilatation is effective for simple benign esophageal stenosis and markedly relieves symptoms in most patients after 1–3 treatments, with 25–35% of patients needing repeated dilatation treatment [11]. Compared with simple stenosis, the efficiency of endoscopic dilatation treatment is considerably worse for complex stenosis, and most patients do not experience relief from symptoms until repeated dilatation treatment is performed; in addition, the rate of recurrence is relatively high [10]. Cicatricial stenosis caused by ESD is mostly refractory, and EBD is the current standard treatment, and it is used as the standard control in other innovative studies [9, 21]. However, repeated dilatation treatment is usually necessary after the occurrence of stenosis to achieve the therapeutic purpose.

Endoscopic dilatation treatment is generally divided into dilatation on demand and dilatation on time. The former refers to dilatation performed when patients develop dysphagia, especially when the dysphagia grade is >2 according to the five-point method [39]. The latter refers to endoscopic dilatation performed on time after ESD, which usually begins on day 3 after ESD at a frequency of twice a week for 8 weeks. If dysphagia persists after 8 weeks, dilatation on time is continued until the symptoms subside. The frequency of balloon dilatation is usually proportionate to the degree of esophageal perimeter mucosal defect and the degree of stenosis. Yamaguchi et al. [9] reported that patients underwent balloon dilatation by a mean of 16 times and that one patient with circumferential lesions underwent dilatation up to 48 times. In addition, studies about preventive balloon dilatation are not rare. Ezoe et al. [40] reported on 41 patients after EMR/ESD with mucosal defects accounting for more than three-fourths of the esophageal lumen perimeter, among whom 29 patients underwent EBD within 1 week at a frequency of once a week until the mucosal defects completely resolved. Twelve previous patients were chosen as the historical blank control group in which routine EBD was conducted when they developed esophageal stenosis until stenosis was corrected. As a result, preventive endoscopic dilatation reduced the incidence and severity of stenosis as well as patients' tolerance to stenosis. In a case report of 2 patients, Wong et al. [41] indicated that early and regular EBD was effective for preventing and treating esophageal stenosis after ESD.

The major complications of endoscopic dilatation treatment include perforation, hemorrhage, and bacteremia. The incidence of perforation and massive hemorrhage was reported to be approximately 0.3% but was considerably higher in cases of complex stenosis or other benign esophageal stenoses [42, 43]. Although few studies have reported the complications of the prevention and treatment of stenosis after ESD, it is generally accepted that the risk of perforation can be significantly reduced if the dilatation diameter is increased by ≤3 mm each time. The diameter and length of esophageal stenosis before dilatation are key factors that affect the required dilatation efficiency and frequency.

### 3.2. Use of Stents

Esophageal metallic stents were initially used in the minimally invasive treatment of esophageal fistula and unresectable malignant esophageal stenosis with common complications and adverse effects such as granulation tissue hyperplasia, pain, stent displacement, and esophageal ulcers [44–46]. With the development of removable temporary coated metallic stents and plastic stents in recent years, stent implantation has gradually become a new treatment option for refractory benign esophageal stenosis [47–49]. Various stents have been used in clinical practice, including recyclable coated metallic stents, recyclable coated plastic stents, drug-eluting stents, antidisplacement stents, and biodegradable stents. However, not all types of stents can be used in patients with stenosis due to the characteristics of esophageal stenosis after ESD. Combined with the application and related reports of stents used for the treatment of esophageal stenosis after ESD in recent years, we summarized several methods as follows.

*Temporary Self-Expandable Metallic Stents*. The major advantage of these stents in the treatment of benign esophageal stenosis is that they can provide a sustained dilatation effect to the stenosed segment and can be removed when the stenosis is relieved or when complications occur. Several recent studies have reported the use of temporary self-expandable metallic stents to treat esophageal benign stenosis [47, 50, 51], which show that stent implantation is effective to some extent in the treatment of stenosis and can alleviate the symptoms in some patients. Nevertheless, some studies have found that the long-term effect of temporary self-expandable metallic stents after implantation was not as satisfactory as expected and that the incidence of complications such as granulation tissue hyperplasia, chest pain, and stent displacement was relatively high [47, 52]. As for the treatment of stenosis after ESD, Matsumoto et al. [53] reported a patient who developed dysphagia 1 month after ESD for squamous cell carcinoma. To treat the endoscopically visible cicatricial stenosis, bougienage was performed once a week and then reduced to once every 2 weeks 1 month later for 15 dilatations. Because the efficiency was unsatisfactory, a temporary metallic stent was implanted and then removed 1 week later. The patients did not present with complications such as chest pain or fever, and no recurrence of stenosis or esophageal mucosal damage was observed during gastroscopy 1 month later. In contrast, Wen et al. [54] found that covered esophageal stent placement for the prevention of esophageal strictures after ESD is effective and safe. In their random control test, the fully covered esophageal stent was placed immediately after ESD at the site of the peeling surface and then removed 8 weeks later. They concluded that the proportion of patients who developed a stricture was significantly lower in the stent group than in the control group. Moreover, the number of bougie dilatation procedures was significantly lower in the stent group than in the control group.

*Biodegradable Stents*. Because of the various drawbacks of metallic and plastic stents, some researchers have used biodegradable stents to treat benign esophageal stenosis. Japanese researchers Tanaka et al. [55] were the first to use polylactide biodegradable stents in 2 patients with esophageal stenosis and obtained promising results. Similarly, in the study by Saito et al. [56], the stents were used in 2 patients who developed stenosis after ESD for early esophageal cancer. The mucosal defects accounted for 7/8 of the esophageal perimeter in both patients. Polylactide biodegradable stents were implanted after balloon dilatation when the stenosis occurred, and no adverse effects or recurrence was observed in the 6 months after implantation.

*Extracellular Matrix Stents*. In a dog model, Badylak et al. [57] found that extracellular matrix stents combined with autologous muscle tissues allowed reconstruction of esophageal structure and recovery of function without the formation of cicatricial stenosis. The extracellular matrix was first prepared by the processing of a pig bladder, made into pipe shapes after decellularization and sterilization, and finally used in esophageal reconstruction as biodegradable stents. Another dog model was established in 2009 by Nieponice et al. [58] in which extracellular matrix stents were used to prevent esophageal stenosis after circumferential EMR. In that study, extracellular matrix stents were implanted through endoscopy in 5 dogs after EMR using another 5 dogs as blank controls. As a result, none of the dogs in the treatment group presented with esophageal stenosis and no significant cicatrices or inflammation was observed in the pathological specimens. In contrast, esophageal stenosis occurred in all 5 dogs in the control group and epithelialization and incomplete inflammation were observed at the EMR site.

## 4. Conclusion

In summary, although many methods are available for the prevention and treatment of esophageal stenosis after ESD, no single method has been widely recognized as effective in clinical practice. Experimental studies have emerged in recent years, but most of them are in the animal research stage. There are sporadic case reports and series studies but there are no randomized controlled trials or systematic reviews with sufficient evidence. However, an accurate preoperative evaluation is essential to fully understand the possibility of postoperative esophageal stenosis and prepare active and effective preventive measures. According to patient and medical conditions, simple but effective preventive measures are crucial to reducing the risk of postoperative esophageal stenosis. Additionally, the feasibility and effectiveness of innovative materials and methods should also be fully affirmed, which should be the focus of future studies. The wide use of these new materials and methods in clinical practice will allow the establishment of more significant conclusions by large sample multicenter randomized controlled trials.
